# Histological and immunohistochemical soft‐tissue response to cylindrical and concave abutments: Multicenter randomized clinical trial

**DOI:** 10.1002/JPER.24-0250

**Published:** 2024-08-26

**Authors:** Fabio Camacho‐Alonso, Juan Carlos Bernabeu‐Mira, Joaquín Sánchez, Antonio Julián Buendía, Ana María Mercado‐Díaz, Mario Pérez‐Sayáns, Alba Pérez‐Jardón, José Manuel Somoza Martín, Javier Montero, Cristina Gomez‐Polo, Norberto Quispe‐López, David Peñarrocha‐Oltra

**Affiliations:** ^1^ Department of Oral Surgery University of Murcia Murcia Spain; ^2^ Oral Surgery and Implant Dentistry, Oral Surgery Unit, Department of Stomatology, Faculty of Medicine and Dentistry University of Valencia Valencia Spain; ^3^ Department of Histology and Pathological Anatomy University of Murcia Murcia Spain; ^4^ Oral Medicine, Oral Surgery and Implantology Unit, Faculty of Medicine and Dentistry. Health Research Institute of Santiago de Compostela, ORALES Group. Materials Institute of Santiago de Compostela Universidad de Santiago de Compostela Santiago de Compostela A Coruña Spain; ^5^ Department of Surgery, Dental Clinic of the Faculty of Medicine University of Salamanca Salamanca Spain

**Keywords:** dental implant‐abutment design, dental implants, histology, immunohistochemistry, multicenter study, oral surgical procedures, randomized controlled trial

## Abstract

**Background:**

This study aimed to analyze the influence of concave and cylindrical abutments on peri‐implant soft tissue. Dimensions, collagen fiber orientation, and immunohistochemical data were assessed.

**Methods:**

A multicenter, split‐mouth, double‐blind randomized clinical trial was conducted. Two groups were analyzed: cylindrical abutments and concave abutments. After a 12‐week healing period, peri‐implant soft tissue samples were collected, processed, and evaluated for dimensions, collagen fiber orientation, and immunohistochemical data. Inflammatory infiltration and vascularization were assessed, and the abutment surfaces were analyzed using scanning electron microscopy. The statistical analysis was performed using the SPSS version 20.0 statistical package.

**Results:**

A total of 74 samples in 37 patients were evaluated. Histological evaluation of peri‐implant soft tissue dimensions revealed significant differences between concave and cylindrical abutments. Concave abutments exhibited greater total height (concave: 3.57 ± 0.28 – cylindrical: 2.95 ± 0.27) and barrier epithelium extension (concave: 2.46 ± 0.17 – cylindrical: 1.89 ± 0.21) (*p* < 0.05), while the supracrestal connective tissue extension (concave: 1.11 ± 0.17 – cylindrical: 1.03 ± 0.16) was slightly greater (*p* > 0.05). Collagen fiber orientation favored concave abutments (23.76 ± 5.86), with significantly more transverse/perpendicular fibers than for cylindrical abutments (15.68 ± 4.57). The immunohistochemical analysis evidenced greater inflammatory and vascular intensity in the lower portion for both abutments, though concave abutments showed lower overall intensity (concave: 1.05 ± 0.78 – cylindrical: 1.97 ± 0.68) (*p* < 0.05). The abutment surface analysis demonstrated a higher percentage of tissue remnants on concave abutments (42.47 ± 1.32; 45.12 ± 3.03) (*p* < 0.05).

**Conclusions:**

Within the limitations of this study, concave abutments presented significantly greater peri‐implant tissue height, linked to an extended barrier epithelium, versus cylindrical abutments in thick tissue phenotype. This enhanced soft tissue sealing, favoring a greater percentage of transversely oriented collagen fibers. The concave design reduced chronic inflammatory exudation with T and B cells, thus minimizing the risk of chronic inflammation.

**Plain Language Summary:**

This study looked at how 2 different shapes of dental implant abutments (the parts that connect the implant to the crown), specifically concave and cylindrical, affect the soft tissue around the implants. We wanted to see how these shapes influenced the tissue's size, structure, and health. We conducted a clinical trial with 37 patients, comparing the 2 types of abutments in the same mouth over 12 weeks.

Our findings showed that the concave abutments led to a taller and more extensive layer of protective tissue around the implant compared to the cylindrical ones. This protective tissue had more favorable collagen fiber orientation, which is important for the strength and health of the tissue. Additionally, the concave abutments resulted in less inflammation and better tissue integration.

In conclusion, concave abutments may provide better support and health for the soft tissue around dental implants, reducing the risk of chronic inflammation and potentially leading to better long‐term outcomes for patients with dental implants

## 1 INTRODUCTION

Ensuring the prevention of marginal bone loss (MBL) is vital to protect rough implant surfaces from exposure to the oral environment. The formation of oral biofilms on these surfaces, coupled with various local and host‐related risk factors, may trigger peri‐implant disease.^1^, ^2^ Multiple factors influencing MBL require comprehensive understanding. An impact of prosthetic abutment design on supracrestal peri‐implant soft tissue and MBL has been suggested.^3^ A recent systematic review and meta‐analysis^4^ demonstrated that narrow abutment designs result in significantly less MBL, though clinically no influence was observed upon the soft tissues.

Four randomized clinical trials have explored prosthetic abutment shape in relation to MBL. Two of the studies^5^, ^6^ found statistically significant differences, while 2 did not.^7^, ^8^ The 2 studies reporting significant differences described more marked transmucosal geometry modifications than the other 2. They both found that narrower and concave abutments afforded better bone behavior. Studies should be made to determine whether these statistically significant changes at the bone level correspond or do not correspond to differences in morphogenesis and inflammation in the peri‐implant soft tissues.

The evaluation of abutment macro‐ and micro‐geometries and their influence on peri‐implant tissue has been a frequently researched topic in recent decades. Mucosal attachment to the implant (mucointegration) consists of a 1.5‐ to 2‐mm high epithelial portion and a 1‐ to 1.5‐mm high connective tissue portion.^9^ The peri‐implant connective tissue surrounding dental implant abutments is postulated to mitigate early bone resorption by hindering the apical migration of inflammatory cells, as reported by Berglundh et al.^10^ and Rodríguez et al.^11^ Preclinical in vivo research in implant dentistry serves to explore proof‐of‐principle concepts, biological mechanisms, and potential adverse reactions prior to clinical testing. However, human models are less frequently used, due to limitations in tissue preservation.

The primary aim of this randomized clinical trial was to assess the influence of 2 different abutment shapes (cylindrical and concave) on the peri‐implant soft tissue through histological and immunohistochemical analyses. The null hypothesis tested was that prosthetic abutment shape does not significantly influence the peri‐implant soft tissues.

## 2 MATERIALS AND METHODS

### 2.1 Study design

A multicenter, split‐mouth, double‐blind randomized clinical trial with 2 parallel experimental arms was carried out at the University of Murcia, the University of Valencia, the University of Salamanca, and the University of Santiago de Compostela (Spain). The study protocol was approved by the Institutional Review Board of the University of Valencia (Ref.: H1524219380739), the Galician Ethics Committee for Clinical Studies (Ref.: 2021/449), and the Ethics Committee of the University of Murcia (3598/2021). The principles outlined in the Declaration of Helsinki on clinical research involving human subjects were followed. Written informed consent was obtained from each enrolled patient. This clinical trial was recorded on *ClinicalTrials.gov* with registration number: NCT03888339, and is reported according to the CONSORT guidelines.^12^

**FIGURE 1:**
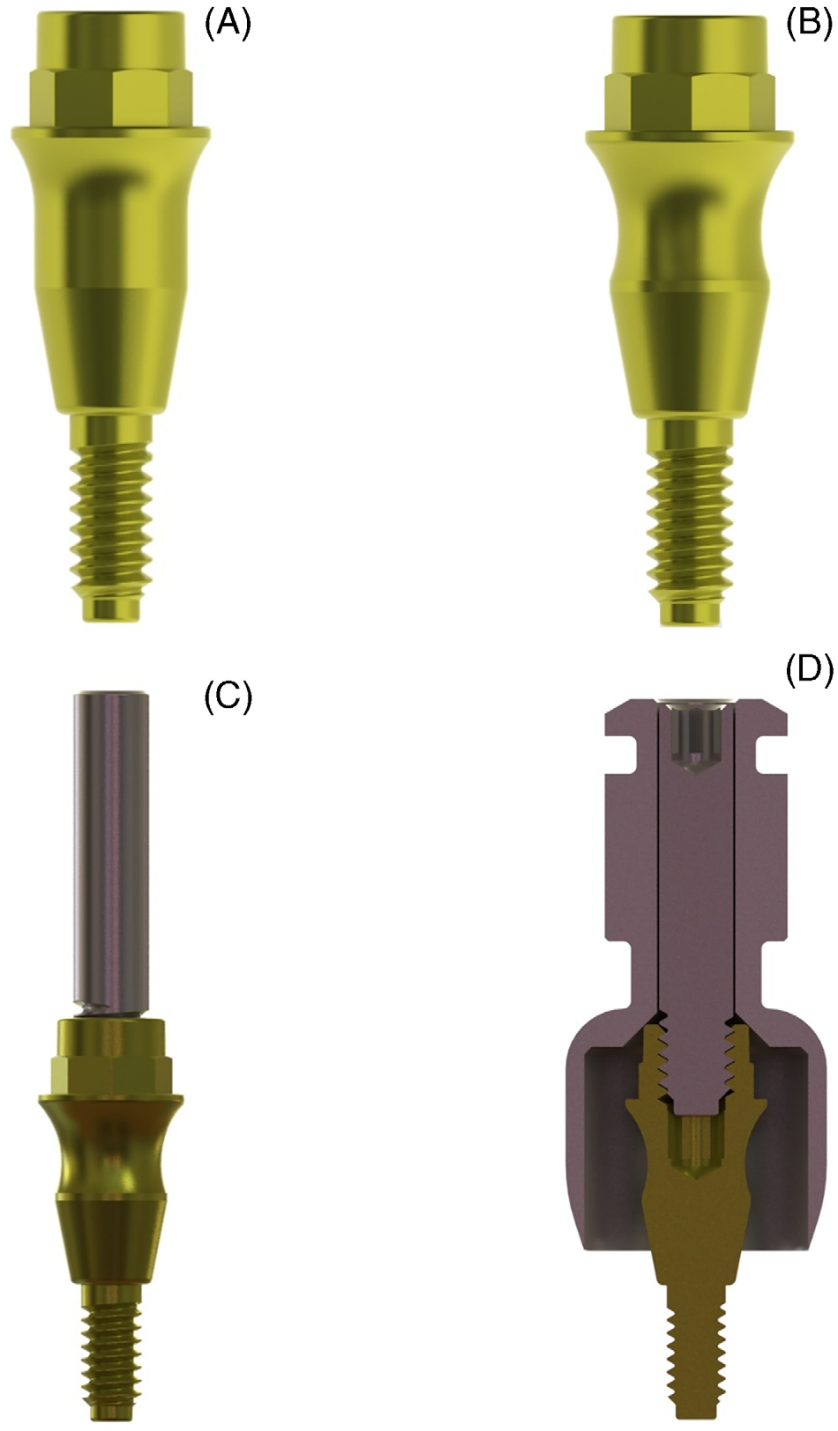
Abutments and circular punch used. (A) Cylindrical abutment. (B) Concave abutment. (C) Guide pin connected to each abutment. (D) Circular punch positioned apically around the abutment

Two study groups were established (Figure 1):

1. Cylindrical group (Figure 1A): 3‐mm high abutments (rotational aesthetic straight abutment,*), referred to as “cylindrical abutments”.
2. Concave group (Figure 1B): 3‐mm high abutments (rotational slim abutment,*), referred to as “concave abutments”.

Both groups shared the same height and connection design, including the presence of a switching platform, and the same surface treatment (machined titanium with anodization).

### 2.2 Study participants

The inclusion criteria were: partially edentulous patients needing 2 implants in the posterior mandible or maxilla (interdental space or free distal part); age ≥ 18 years; absence of systemic conditions unsuitable for implant surgery (e.g., immunosuppression, head/neck irradiation, intravenous amino‐bisphosphonate treatment, uncontrolled diabetes, pregnancy, or psychiatric issues). The patients were required to be non‐smokers or smokers of ≤10 cigarettes/day; periodontally healthy with a Silness and Löe plaque index^13^ and bleeding on probing score of <25% (when the tooth was positive in any of the values 1–2, the tooth was considered positive. If 25% of the teeth bled, the patient was excluded); average of periodontal pockets ≤ 3 mm; and healed bone for at least 3 months following extraction, with no infection. Adequate bone volumes allowing for implant placement (8–14 mm length and 4, 4.5, and 5 mm width) without regeneration procedures were required, as well as a minimum vertical supracrestal soft tissue thickness of 3 mm, and at least a 2 mm width of keratinized mucosa around the abutment. Patients unable to complete the follow‐up, presenting implant failures, or with primary implant stability <35 Ncm were excluded from the study.

### 2.3 Interventions

#### 2.3.1 Screening visit

Potentially eligible patients underwent screening, which included a review of the clinical history, anamnesis, oral examination, preoperative panoramic radiography, and cone‐beam computed tomography (CBCT). The CBCT scan involved the use of 2 cotton swabs to separate the tongue and buccal mucosa from the edentulous area, facilitating the measurement of soft tissue thickness. An oral hygiene session was arranged within 7 days before the implant placement procedure. A total of 2 g of amoxicillin p.o. was prescribed 1 hour before the intervention as prophylaxis.

#### 2.3.2 Implant placement

Local anesthesia involved the administration of 4% articaine with 1/200,000 adrenaline†. A crestal incision was made, a buccal flap was raised, and implants were placed in equicrestal positions. Galimplant IPX implant* (bone‐level design with an internal conical connection) were placed. Implant sizes were 4, 4.5, or 5 mm in diameter and 8–14 mm in length, tailored to the available bone. The healing process for the implants was conducted based on a non‐submerged technique. As a postoperative medication, 1 g of paracetamol every 8 h was prescribed on demand. Postoperative hygiene and oral care instructions were explained to the patient. A mouthwash consisting of 0.12% chlorhexidine twice daily for 2 weeks was also recommended.

#### 2.3.3 Abutment placement

When the 2 implants of the patient were placed with at least 35 Ncm of primary stability, the prosthetic abutments were intraoperatively screw‐retained at 30 Ncm. The randomization and allocation processes were performed after implant placement and before abutment placement. The 2 types of abutments (cylindrical [Figure 1A] and concave [Figure 1B]) were placed in the same patient. Implant stability quotient (ISQ) measurements were made after abutment placement. Regarding anodization, the 2 abutments were subjected to the same surface treatment. The anodization procedure was performed by cleaning the surface, eliminating the natural layer of oxide and subsequently immersing the abutment in an electrolytic cell containing a solution based on phosphoric acid at a given energy intensity. At the end of the process, the abutments were rinsed in deionized water for 2 minutes and washed with deionized water in an ultrasonic bath for 20 min.

#### 2.3.4 Biopsy procedure

After the assigned healing time of 12 weeks, an ad hoc guide pin was connected to each abutment (Figure 1C). A circular punch, 6 mm wide and with a cutting edge, was positioned apically around the abutment (Figure 1D). Then, a 1.1–1.7 mm thick collar of peri‐implant soft tissue was dissected and removed together with the abutment, and immersed in a fixative solution of 10% buffered formalin. After biopsy and removal of the punch, a specific ad hoc designed healing plug was placed over the implant to allow adequate sealing and healing.

#### 2.3.5 Sample processing after biopsy

Following fixation, the abutments were removed for subsequent study using a field emission scanning electron microscope (FESEM)‡.

After dehydration in a graded series of ethanol rinses, the collar of peri‐implant soft tissue was embedded in paraffin and sectioned parallel with the long axis of the abutment in order to obtain 6 equal portions: portion 1 (hematoxylin‐eosin and Masson‐Goldner trichrome stain), portion 2 (CD3), portion 3 (CD79a), portion 4 (CD68), portion 5 (anti‐myeloperoxidase), and portion 6 (CD31). Finally, 4‐μm sections were obtained from all the portions, and a single well‐trained examiner not involved in the surgical treatment evaluated the histological and immunohistochemical results. A code was reported on the histological slides in order to blind the abutment features.

#### 2.3.6 Prosthetic treatment

Impressions were taken at the abutment level 2 weeks after the biopsy to create a cemented‐screw retained zirconia prosthesis. All screws were securely tightened at a torque of 25 Ncm.

### 2.4 Outcome measurements

#### 2.4.1 General variables

Patient age and sex, smoking habit (non‐smoker or smoker of ≤10 cigarettes/day), implant site, implant length (8–14 mm), implant diameter (4, 4.5, or 5 mm), insertion torque, and ISQ values were recorded.

#### 2.4.2 Evaluation of the peri‐implant soft tissue: Height dimension (portion 1)

**FIGURE 2:**
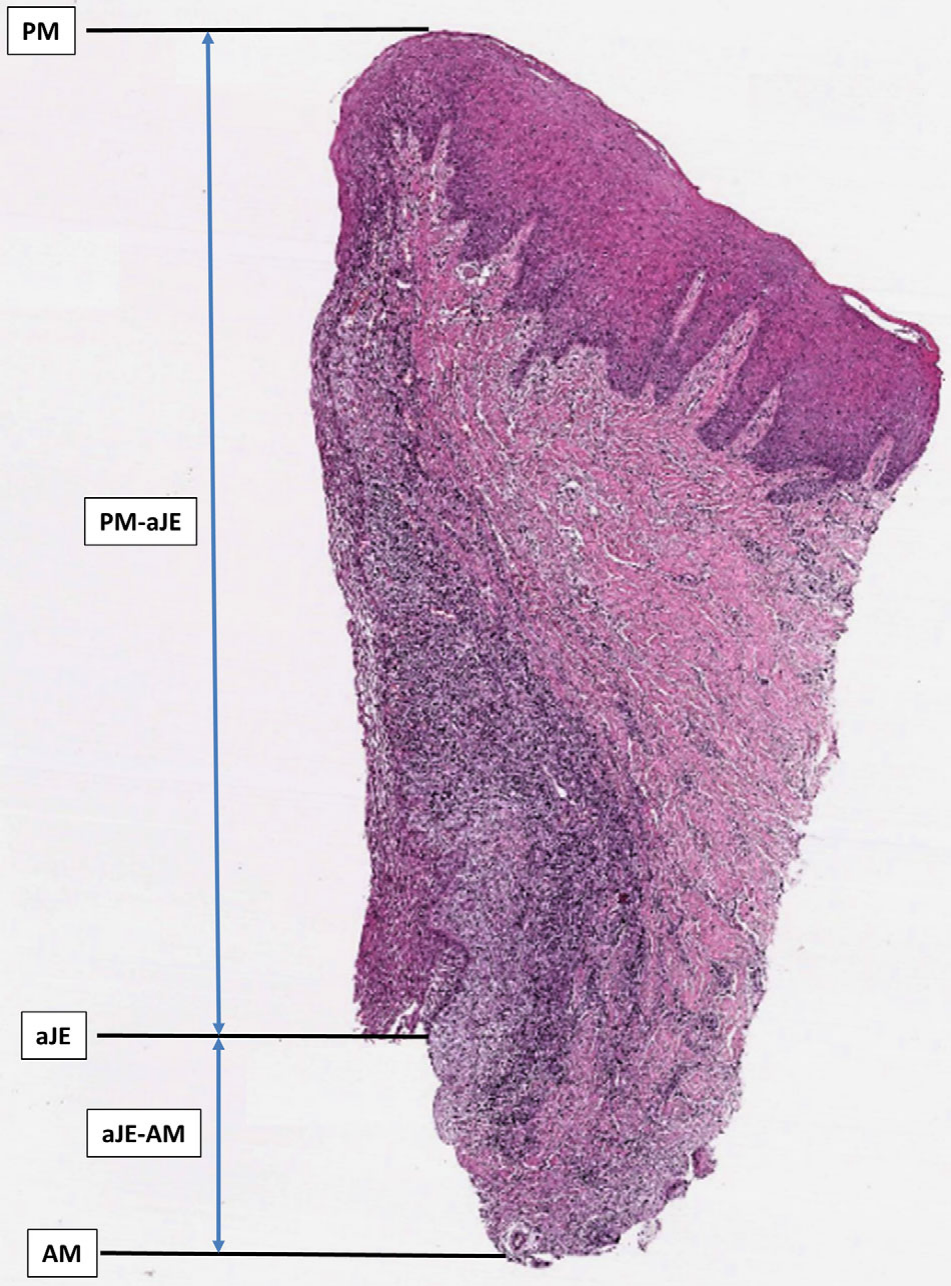
Illustration of the histological measurements used to calculate the peri‐implant soft tissue (height) dimension. AM, apical location of the mucosal part; aJe, apical extension of the barrier epithelium; aJE‐AM, extension of the supracrestal connective tissue; PM, margin of the peri‐implant mucosa; PM‐aJE, extension of the barrier epithelium

Sections stained with hematoxylin‐eosin were used to calculate the peri‐implant soft tissue (height) dimension. The following histological landmarks were identified in each section: the margin of the peri‐implant mucosa (PM), the apical extension of the barrier epithelium (aJE), and the apical location of the mucosal part (AM). The vertical distance was measured in a direction parallel to the long axis of the abutment, following the methodology proposed by Tomasi et al.^14^ and Canullo et al.,^15^ using image analysis software (ImageJ version 1.48v)§ (Figure 2). The histological analysis was carried out under a light microscope (Zeiss Axioscop 40)‖, connected to a high‐resolution video camera (Zeiss Axiocam 503 color)‖ and interfaced with a PC.

#### 2.4.3 Collagen fiber orientation (portion 1)

**FIGURE 3:**
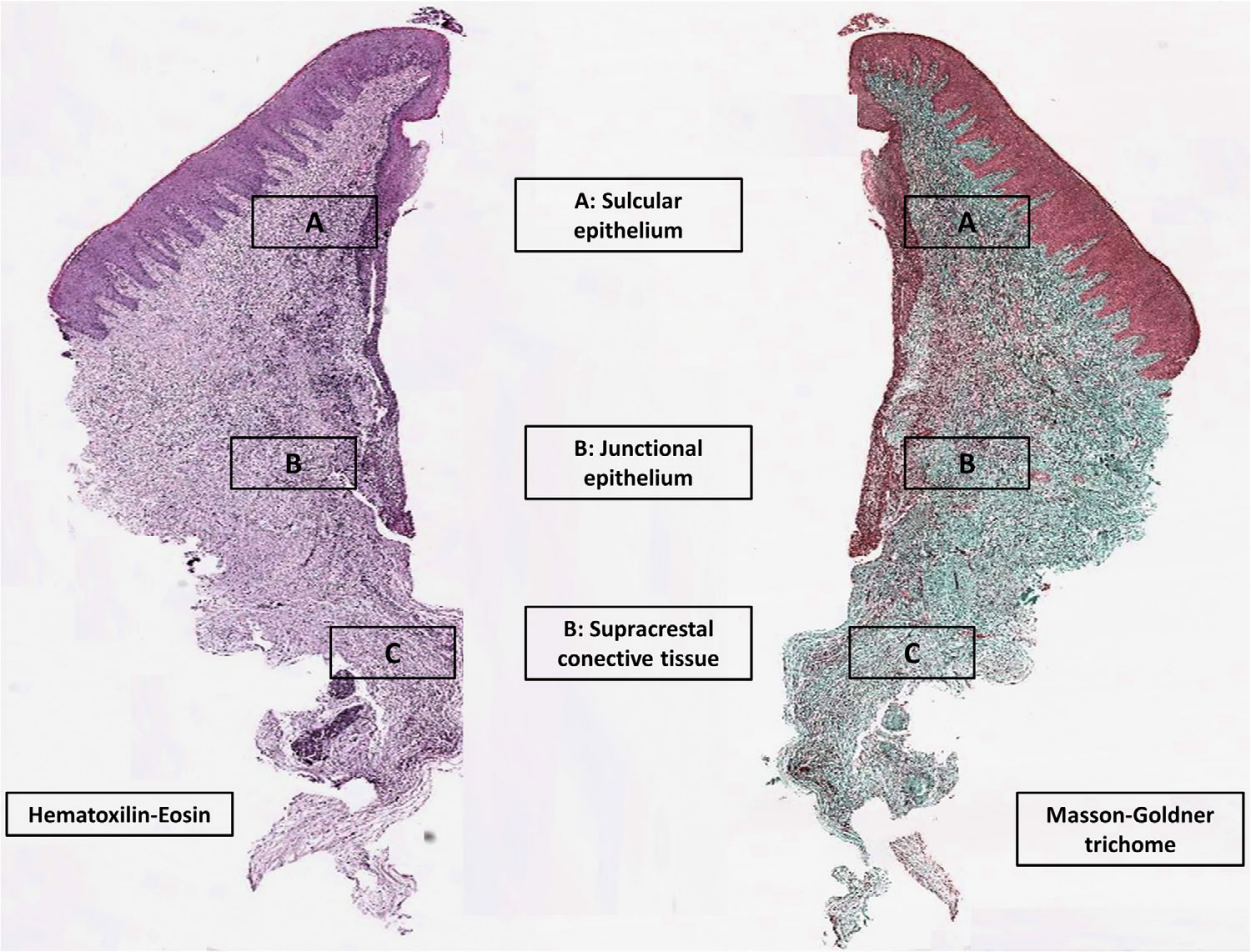
Histological analysis showing 3 ROIs (A, sulcular epithelium; (B, junctional epithelium; and (C, supracrestal connective tissue) used for the study of collagen fiber orientation and immunohistochemical analysis

Sections processed with the Masson‐Goldner trichrome stain were used to calculate collagen fiber orientation. Birefringence allowed highlighting of the orientation using polarized light microscopy (Axiolab)‖ equipped with 2 linear polarizers and 2 quarter‐wave plates arranged for transmission of circularly polarized light. For this purpose, 3 defined regions of interest (ROIs) (sulcular epithelium, junctional epithelium, and supracrestal connective tissue) were analyzed following the technique proposed by Thoma et al.^16^ (Figure 3), determining the total percentage of collagen fibers orientated longitudinal/parallel or transverse/perpendicular to the abutment surface, following the methodology proposed by Valente et al.^3^ and Covani et al.^17^

#### 2.4.4 Immunohistochemical analysis (intensity and composition of the inflammatory exudate and vascular proliferation) (portions 2–6)

Portions 2–6 from the collar of peri‐implant soft tissue were used as follows: portion 2 for T cells (CD3), portion 3 for B cells (CD79a), portion 4 for macrophages (CD‐68), portion 5 for anti‐myeloperoxidase (neutrophil granulocytes), and portion 6 for vessels (CD31). The same region of interest as that used for the collagen fiber orientation study was considered (Figure 3).

Characterization of the inflammatory infiltrate and vascularization was made using the following as primary antibodies: polyclonal rabbit anti‐CD3 (T cells¶), monoclonal mouse anti‐CD79a (B cells#), monoclonal mouse anti‐CD68 (macrophages¶), polyclonal rabbit anti‐myeloperoxidase (neutrophil granulocytes,¶), and monoclonal mouse anti‐CD31 (endothelial cells¶). The immunohistochemical procedure was performed on 3‐μm sections from formalin‐fixed and paraffin‐embedded samples. Briefly, after deparaffination and rehydration, a target retrieval procedure was performed by immersion in ethylenediaminetetraacetic acid (EDTA) pH 9.0 solution¶ at 98°C for 30 min. Sections were then incubated with 0.5% H_2_O_2_ in methanol for 20 min at 37°C to block endogenous peroxidase and to prevent nonspecific background interference (normal horse serum,**). The sections were then further incubated with primary antibody for 1 hour at 37°C, washed in Tris‐buffered saline (TBS), and incubated with the ImmPRESS horse anti‐mouse or anti‐rabbit immunoglobulin G (IgG) polymer kit, peroxidase (Vector for 30 min at 37°C). Finally, the immune reaction was revealed by incubation with 3‐3´ diaminobenzidine (DAB) commercial solution (Dako) for 5 min at room temperature. Positive reactions were identified by a dark brown precipitate. The sections were finally hematoxylin counterstained, dehydrated, cleared, and mounted.

**FIGURE 4:**
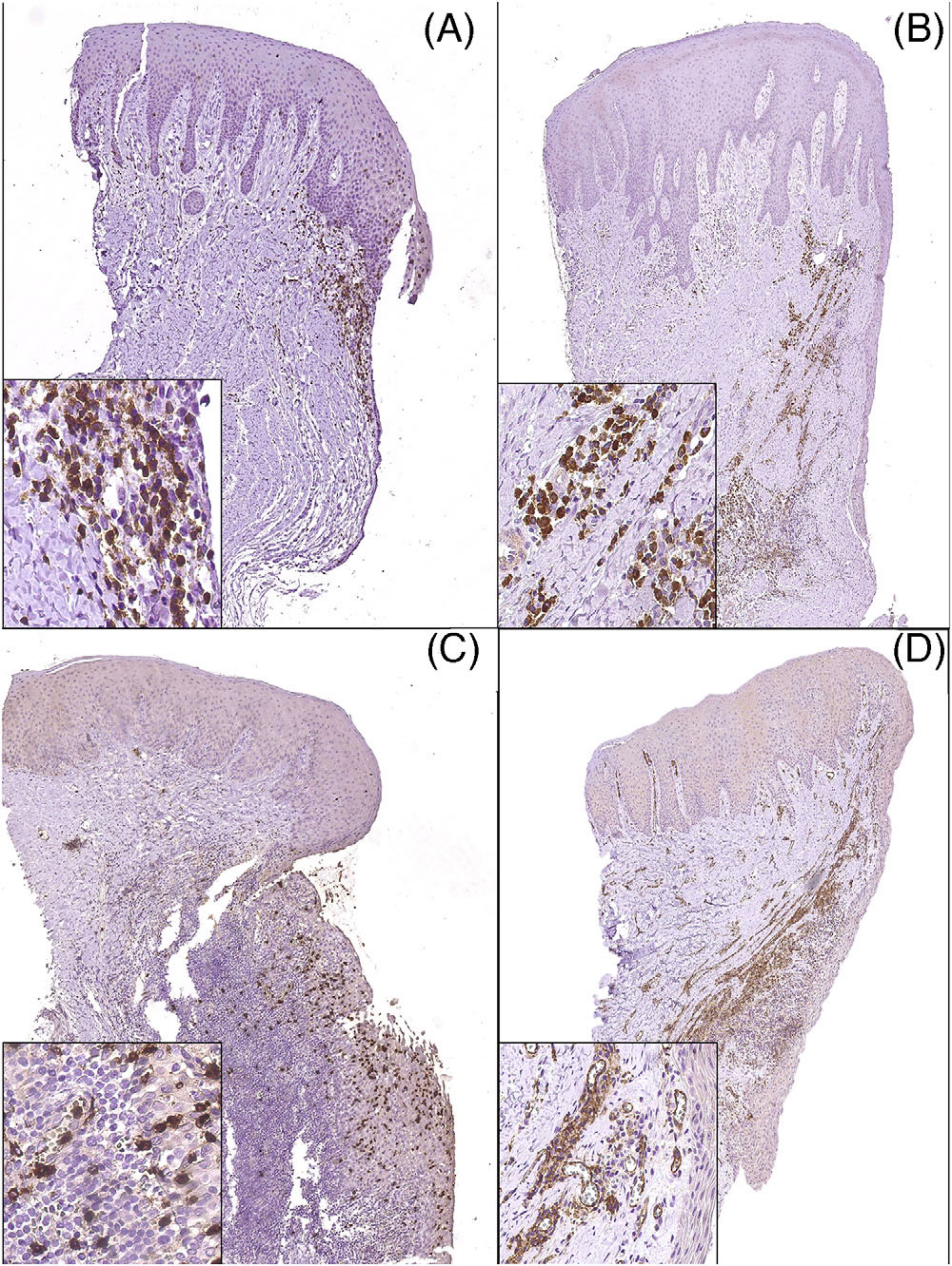
Intensity and composition of the inflammatory exudate and vascular proliferation. (A) Representative image of a grade 1 inflammatory reaction in the gingiva. In this image T cells have been labeled by immunohistochemistry. A detail of immunolabeled T cells (dark brown) can be seen in the inset box. Original magnification: ×50, insert: ×400. (B) Representative image of a grade 2 inflammatory reaction in the gingiva. In this image B cells have been labeled by immunohistochemistry. A detail of immunolabeled B cells (dark brown) can be seen in the inset box. Original magnification: ×50, insert: ×400. (C) Representative image of a grade 3 inflammatory reaction in the gingiva. In this image polymorphonuclear cells have been labeled by immunohistochemistry. A detail of immunolabeled polymorphonuclear cells (dark brown) can be seen in the inset box. Original magnification: ×50, insert: ×400. (D) Representative image of a grade 3 inflammatory reaction in the gingiva. In this image endothelial cells have been labeled by immunohistochemistry. A detail of immunolabeled endothelial cells (dark brown) can be seen in the inset box. Original magnification: ×50, insert: ×400

Based on the intensity and composition of the inflammatory exudate and the vascular proliferation, inflammation was assessed by semiquantitative analysis using a scale from 0 to 3: score 0 (absence of inflammatory cells), score 1 (scarce mixed inflammatory cells, mainly T and B cells, corresponding to mild chronic inflammation) (Figure 4A), score 2 (moderate mixed inflammatory infiltrate composed of T and B cells, with scarce macrophages and polymorphonuclear neutrophils, corresponding to intermediate chronic inflammation, with mild vascular proliferation) (Figure 4B), and score 3 (numerous T and B cells and some macrophages, with abundant polymorphonuclear neutrophils, corresponding to severe chronic‐reactive inflammation, with exuberant vascular proliferation) (Figure 4C,D).

#### 2.4.5 Abutment surface analysis

**FIGURE 5:**
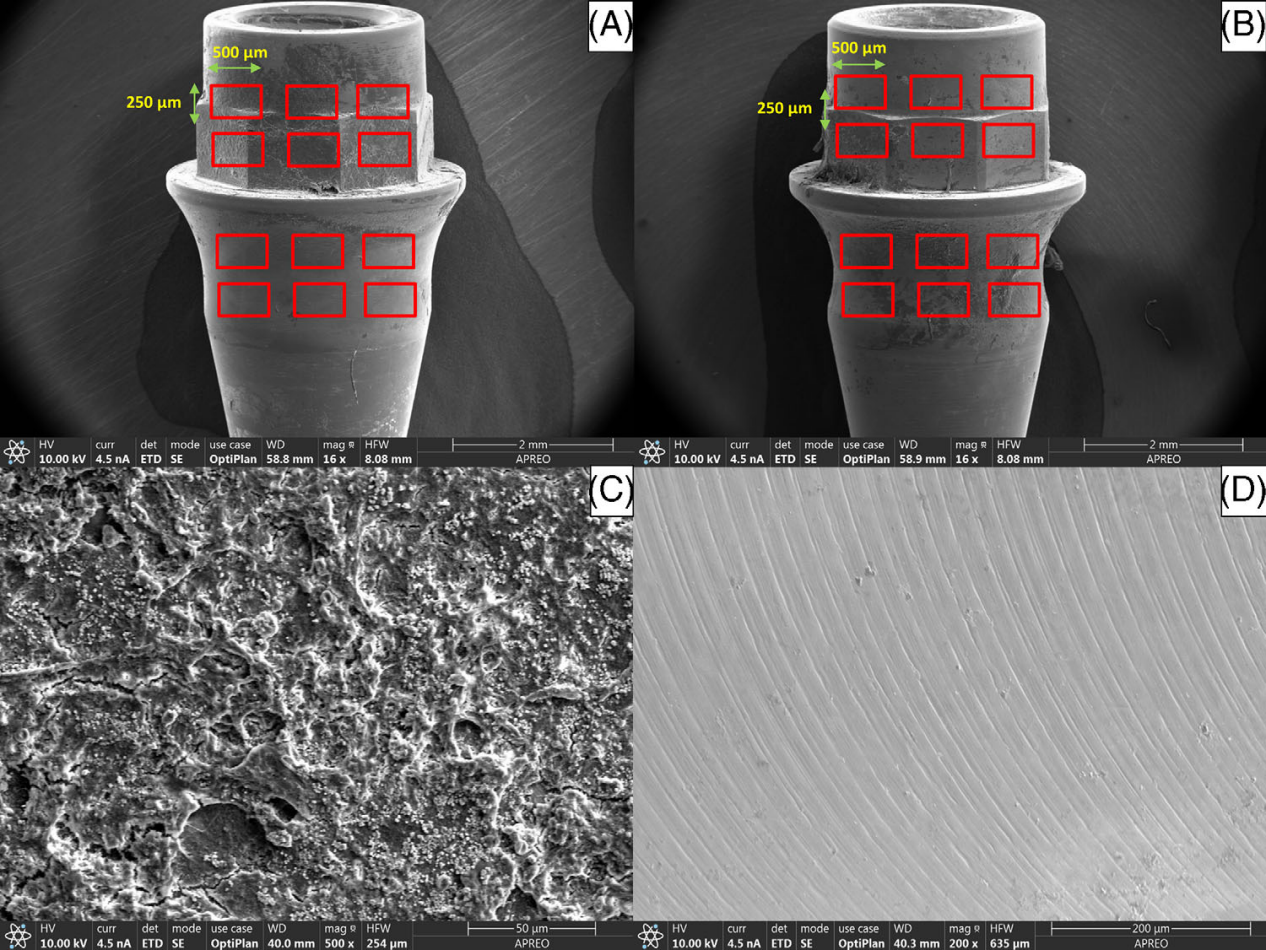
FESEM study. (A) ROIs in cylindrical abutment, magnification: 16x. (B) ROIs in concave abutment, magnification: 16x. (C) Biofilm, magnification: 500x. (D) Titanium, magnification: 200x.

The retrieved abutments were analyzed by FESEM‡. Each abutment was washed twice in phosphate buffered saline (PBS) (pH 7.2), fixed with 1% osmium tetraoxide†† for 2 hours, and washed in 0.1 M sodium cacodylate buffer with sucrose††. Then, the samples were dehydrated by immersion in acetonitrile solutions†† of increasing concentrations (50%, 70%, 80%, 90%, and 100%) twice for 20 min, before critical point drying over metallic platens using a Leica EM CPD030‡‡. Finally, they were platinum sputtered with a 5.0 nm layer using a Leica ACE600‡‡ and examined by FESEM‡, with a voltage setting of 5 kV beam energy, collecting the backscattered electrons signal (BSE) to obtain a compositional contrast between the titanium surface (high atomic number and elevated BSE signal) and biological remnants (low atomic number and reduced BSE signal), as described by Bressan et al.^18^ Twelve ROIs of each abutment (6 below and 6 above the platform) were established based on a standardized study design: each ROI was determined following a grid and rules formerly decided by the researcher, eliminating the bias caused by investigator behavior. The method allowed repeated observations with 100% repeatability in findings in the same observations fields when a 500 × 250 μm specification was adopted (Figure 5A,B). Following the methodology proposed by Tomasi et al.,^9^ the percentages of submucosal abutment surface covered by biofilm (Figure 5C), clean surface (titanium) (Figure 5D) or tissue remnants were analyzed using digitalized images and MIP‐4 histomorphometry software§§. For tissue remnants, the criteria proposed by Dorkan et al.^19^ for scanning electron microscopy (SEM) were used: keratinocytes were defined as clusters of cells with a typical polygonal morphology, spread evenly over the surface, and with few or no filopodia; and fibroblasts were defined as cells with a characteristic elongated morphology, flattened aspect, and abundant filopodia.

#### 2.4.6 Implant success

Assessment of the implants followed the criteria outlined by Misch et al.,^20^ categorizing them into success (optimal health), satisfactory survival, compromised survival, or failure. According to Misch et al.,^20^ success was defined by specific conditions: no pain or tenderness during function, zero mobility, radiographic bone loss less than 2 mm since initial surgery, and no history of exudate. Satisfactory survival meant no pain during function, zero mobility, radiographic bone loss between 2 and 4 mm, and no history of exudate. Compromised survival in turn involved tenderness during function, zero mobility, radiographic bone loss exceeding 4 mm (but less than half the implant length), probing depth over 7 mm, and a potential history of exudates. Last, failure was diagnosed if any of the following were present: pain during function, mobility, radiographic bone loss surpassing half the implant length, uncontrolled exudate, and absence from the oral cavity.

### 2.5 Study population

#### 2.5.1 Sample size calculation

The study population consisted of a minimum of 74 implants in 37 patients (baseline study). The sample size was calculated to obtain differences in fiber thickness of 30 μm between groups, with a power of 80% and an α level of 5%. This calculation was carried out with Epidat 4.2 (SERGAS, Galicia, Spain).

#### 2.5.2 Randomization, allocation, and blinding

Each patient contributed 2 implants to the study. Randomization was performed through www.randomization.com. The random allocation codes were sealed in sequentially numbered opaque envelopes. Allocation concealment was broken after the implant insertions at 35 Ncm, when the corresponding envelope was opened and the operator was informed whether to place cylindrical abutments or concave abutments. Blinding was maintained for the patients and the statistician, and not for the examiner of the samples.

#### 2.5.3 Sample

A total sample of 74 dental implants (*n*) were placed in 37 patients (26 men and 11 women; mean age of 64.38 ± 13.72). The study was conducted from March 2019 to June 2022. All the implants showed success according to the classification of Misch et al.^20^ The preoperative mucosal dimensions from the CBCT were 3.79 ± 1.25 mm (cylindrical group (3.85 ± 1.31) and 3.74 ± 1.20). The homogeneity between both groups is shown in Table S1 in the online Journal of Periodontology.

### 2.6 Statistical analysis

Data were analyzed using the SPSS version 20.0 statistical package‖‖. A descriptive study was made of each variable. Associations between different qualitative variables were analyzed using Pearson's chi‐squared test. The Student *t*‐test for 2 dependent samples was used in application to quantitative variables, in each case determining whether the variances were homogeneous. Differences were regarded as significant if the probability value was *p* ≤ 0.05.

## 3 RESULTS

### 3.1 Evaluation of peri‐implant soft tissue (height) dimension

**FIGURE 6:**
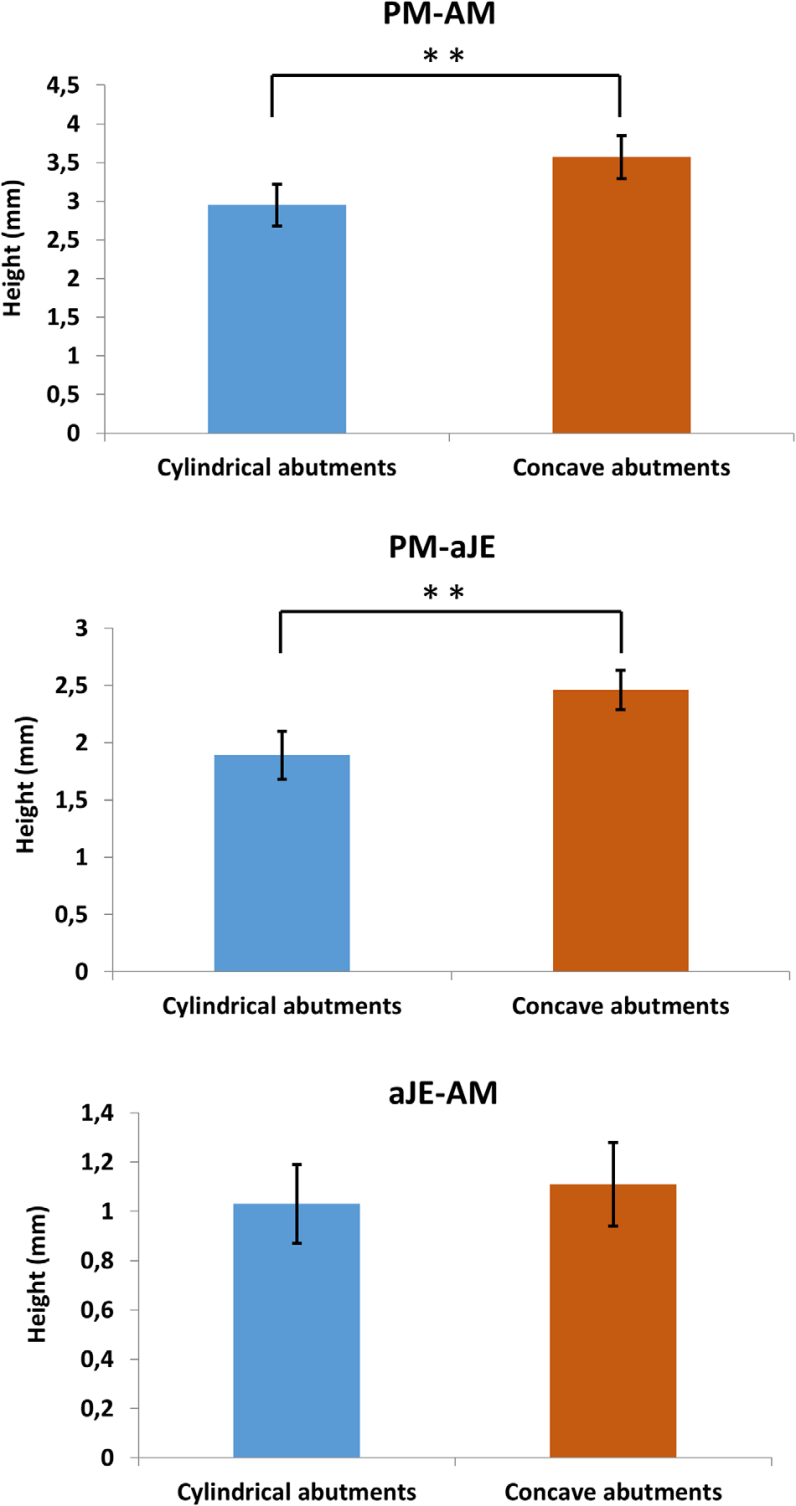
Histomorphometric evaluation of peri‐implant soft tissue height (*p ≤ 0.05, **p ≤ 0.001).

The results from the histological evaluation of the peri‐implant soft tissue (height) dimension are presented in Figure 6. The peri‐implant soft tissue around the concave abutments showed a significantly greater total height (PM‐AM) (3.57 ± 0.28 [range 2.90–4.20]) than around the cylindrical abutments (2.95 ± 0.27 [range 2.10–3.60]) (*p* < 0.001). Likewise, the extension of the barrier epithelium (PM‐aJE) around the concave abutments was significantly greater (2.46 ± 0.17 [range 1.90–2.80]) than with the cylindrical abutments (1.89 ± 0.21 [range 1.40–2.30]) (*p* < 0.001). Last, although the extension of the supracrestal connective tissue (aJE‐AM) was slightly greater around the concave abutments (1.11 ± 0.17 (range 0.90–1.60) than around the cylindrical abutments (1.03 ± 0.16 [range 0.70–1.50]), the difference failed to reach statistical significance (*p* = 0.069).

### 3.2 Collagen fiber orientation

Although the percentage of collagen fibers orientated longitudinally/parallel to the abutment was greater in both types of abutments (84.32 ± 4.57 for cylindrical abutments and 76.24 ± 5.86 for concave abutments), the percentage of fibers orientated transversely/perpendicular to the abutment was significantly greater in the concave abutments (23.76 ± 5.86) than for the cylindrical abutments (15.68 ± 4.57) (*p* < 0.001).

### 3.3 Immunohistochemical analysis (intensity and composition of the inflammatory exudate and vascular proliferation)

In both types of abutments, the greatest inflammatory intensity and vascular proliferation were observed in the lower portion of the barrier epithelium and supracrestal connective tissue.

Inflammatory intensity and vascular proliferation were significantly lower in the peri‐implant soft tissue around the concave abutments (1.05 ± 0.78) than around the cylindrical abutments (1.97 ± 0.68) (*p* < 0.001).

### 3.4 Abutment surface analysis

Respectively for cylindrical and concave abutments, in relation to the percentages of submucosal abutment surface covered by biofilm (2.40 ± 0.44; 1.91 ± 0.48), clean surface (55.17 ± 1.07; 52.35 ± 0.65) and surface covered by tissue remnants (42.47 ± 1.32; 45.12 ± 3.03), the concave abutment surface showed a significantly greater percentage of tissue remnants than the cylindrical abutment surface (*p* = 0.040), but not for the other 2 variables.

## 4 DISCUSSION

Concave abutments exhibited greater total height and barrier epithelium extension than cylindrical abutments. Collagen fiber orientation favored the concave abutments, with significantly more transverse/perpendicular fibers. The immunohistochemical analysis evidenced greater inflammatory intensity and vascular proliferation in the lower portion for both abutments, though the concave abutments showed lower overall intensity. The abutment surface analysis likewise demonstrated a greater percentage of tissue remnants on the concave abutments.

In contrast to our findings, a recent study^15^ detected no statistically significant differences in the height of the epithelium. The possible reason for this, as mentioned by the latter authors, was the identical length of the different abutments evaluated. Their study used straight truncated cone profiles, ensuring the same vertical height of the abutments. Concave abutments increase the apico‐coronal distance of the abutment surface without increasing the height – a viable choice in anatomically compromised situations. Accordingly, these macro‐geometric modifications increase barrier epithelium extension.

A recent preclinical study^17^ revealed that a concave transmucosal design had the potential to enhance connective tissue deposition and growth compared to a straight transmucosal design. An increase in connective tissue, thickening of the peri‐implant network, and alignment of the collagen fibers towards the abutment collar, forming a broad circular collagen structure around the implant platform, were detected. The results of the present study are consistent with these findings. The introduction of a concave profile resulted in the organization of the collagen fibers into abundant parallel bundles.

The orientation of the collagen fibers plays a crucial role in biomechanics, as it serves as an indication of the forces exerted upon the connective tissue, specifically by the collagen bundles. Several studies have emphasized that a large quantity of collagen, lacking directionality, leads to the formation of dysfunctional fibrotic structures.^21^, ^22^, ^23^ This orientation provides essential insights into the stability of the peri‐implant soft tissue, as highlighted by Karjalainen et al.^24^ Relying solely on an assessment of connective tissue thickness (whether vertical or transversal) is insufficient to ensure tissue stability.

Two randomized clinical trials^5^, ^6^ recorded statistically significant differences in MBL due to different abutment geometries, with more favorable results in the case of narrower abutments. According to the present study, the aforementioned significant clinical changes at the bone level seem to be histologically and immunohistochemically correlated to the structure of the peri‐implant soft tissues around different prosthetic abutments. Recent hypotheses propose that effectively organized connective tissues around the dental implant neck may mitigate early bone resorption by impeding the apical migration of inflammatory cells.^10^, ^11^

The limitations of the present study were the short follow‐up period involved (3 months), and the 2‐dimensional analysis of the peri‐implant soft tissues. The results obtained should only be considered for subcrestal bone‐level implants with a conical internal connection, platform‐switching, and this specific implant abutment design. All patients were periodontally healthy, with no history of periodontitis, and with abundant bone and soft tissue. The specific patient demographics and center effect was not considered. Further studies are needed to understand the behavior of other abutment designs. Patient‐reported outcomes may be considered for future investigations.

## 5 CONCLUSIONS

Within the limitations of this study, concave abutments presented significantly greater peri‐implant tissue height, linked to an extended barrier epithelium, compared to cylindrical abutments in thick tissue phenotype. This enhances soft tissue sealing, promoting a greater percentage of transversely oriented collagen fibers. The concave design reduces chronic inflammatory exudation with T and B cells, minimizing the risk of chronic inflammation.

## AUTHOR CONTRIBUTIONS

Fabio Camacho‐Alonso: Research design and methodology, data analysis and interpretation, and manuscript writing and critical revision. Juan Carlos Bernabeu‐Mira: Contributed to the experimental design, data collection and analysis, and manuscript drafting and review. Joaquín Sánchez, Antonio Julián Buendía, and Ana María Mercado‐Díaz: Involved in data collection and experimental procedures and contributed to literature review. Mario Pérez‐Sayáns: Manuscript writing and critical review. Alba Pérez‐Jardón and José Manuel Somoza Martín: Contributed to clinical aspects of the study and provided expertise in a particular field (oral surgery). Javier Montero: Involved in data analysis and interpretation and manuscript drafting and review. Cristina Gomez‐Polo: Contributed to the experimental design, data analysis and interpretation, and manuscript writing and critical review. Norberto Quispe‐López: Involved in experimental procedures and contributed to data analysis and interpretation. David Peñarrocha‐Oltra: Contributed to the study design and methodology and manuscript drafting and review.

## FUNDING INFORMATION

The authors received no specific funding for this work.

## CONFLICT OF INTEREST STATEMENT

The authors declare no conflicts of interest.

## Supporting information

Supporting Information

Supporting Information

## Data Availability

The data are available upon request from the authors.

## Footnotes

* Nueva Galimplant SLU, Sarria, Galicia, Spain.

† Septadent, Septodont, Madrid, Spain.

‡ ApreoS Lovac IML, Thermo Fisher Scientific, Waltham, MA, USA.

§ National Institutes of Health, MD, USA.

‖ Zeiss, Oberkochen, Germany.

¶ Agilent, Santa Clara CA, USA.

# Bio‐Techne, Minneapolis MN, USA.

** Vector Laboratories, Burlingame, CA, USA.

†† Sigma–Aldrich Chemistry, S.A., Madrid, Spain.

‡‡ Leica Microsystems, Heidelberg GmbH, Mannheim, Germany.

§§ Digital Image, Barcelona, Spain.

‖‖ SPSS Inc., Chicago, IL, USA.
