# Supplementary material for: Histological and immunohistochemical soft‐tissue response to cylindrical and concave abutments: Multicenter randomized clinical trial
Source: J Periodontol. 2024 Aug 26;96(5):418–28. doi: 10.1002/JPER.24-0250 (PMC12123392; doi:10.1002/JPER.24-0250)
Supplement: Supplementary file 1 — Supporting Information [file JPER-96-418-s001.pdf]

Supplementary Material 1: Homogeneity of the study groups in terms of the sites, length, diameter, insertion torque and ISQ values (*Pearson's* χ^2 and *Student t* tests).

**Characteristics**	**Cylindrical abutments (n=37)**	**Concave abutments (n=37)**	***p-value***
**Site:**	n (%)		0.484
4	1 (2.71)	3 (8.11)	
5	2 (5.41)	2 (5.41)	
6	4 (10.81)	2 (5.41)	
7	2 (5.41)	1 (2.71)	
12	2 (5.41)	1 (2.71)	
13	0 (0)	4 (10.81)	
14	4 (10.81)	3 (8.11)	
15	0 (0)	3 (8.11)	
21	2 (5.41)	0 (0)	
20	3 (8.11)	3 (8.11)	
19	5 (13.51)	4 (10.81)	
18	4 (10.81)	3 (8.11)	
28	0 (0)	1 (2.71)	
29	1 (2.71)	2 (5.41)	
30	5 (13.51)	2 (5.41)	
31	2 (5.41)	3 (8.11)	
**Length:**	n (%)		0.795
8 mm	10 (27.03)	12 (32.43)	
10 mm	21 (56.75)	17 (45.94)	
12 mm	5 (13.51)	6 (16.22)	
14 mm	1 (2.71)	2 (5.41)	
**Diameter:**	n (%)		0.835
4.0 mm^2	8 (21.62)	6 (16.22)	
4.5 mm^2	26 (70.27)	28 (75.67)	
5.0 mm^2	3 (8.11)	3 (8.11)	
**Insertion torque (N/cm):** mean ± SD*	41.38 ± 9.41	39.19 ± 9.61	0.990
**ISQ (values):** mean ± SD	73.83 ± 9.76	72.08 ± 9.81	0.447
Preoperative mucosal dimensions: mean ± SD	3.85 ±1.31	3.74±1.20	0.762

* **SD = standard deviation**
